# High diversity, abundance, and expression of hydrogenases in groundwater

**DOI:** 10.1093/ismeco/ycae023

**Published:** 2024-02-12

**Authors:** Shengjie Li, Damon Mosier, Angela Kouris, Pauline Humez, Bernhard Mayer, Marc Strous, Muhe Diao

**Affiliations:** Department of Earth, Energy and Environment, University of Calgary, Calgary, AB T2N 1N4, Canada; Department of Biogeochemistry, Max Planck Institute for Marine Microbiology, Bremen 28359, Germany; Department of Earth, Energy and Environment, University of Calgary, Calgary, AB T2N 1N4, Canada; Department of Earth, Energy and Environment, University of Calgary, Calgary, AB T2N 1N4, Canada; Department of Earth, Energy and Environment, University of Calgary, Calgary, AB T2N 1N4, Canada; Department of Earth, Energy and Environment, University of Calgary, Calgary, AB T2N 1N4, Canada; Department of Earth, Energy and Environment, University of Calgary, Calgary, AB T2N 1N4, Canada; Department of Earth, Energy and Environment, University of Calgary, Calgary, AB T2N 1N4, Canada

**Keywords:** groundwater, microorganisms, hydrogenase, metagenomics, proteomics

## Abstract

Hydrogen may be the most important electron donor available in the subsurface. Here we analyse the diversity, abundance and expression of hydrogenases in 5 proteomes, 25 metagenomes, and 265 amplicon datasets of groundwaters with diverse geochemistry. A total of 1545 new [NiFe]-hydrogenase gene sequences were recovered, which considerably increased the number of sequences (1999) in a widely used database. [NiFe]-hydrogenases were highly abundant, as abundant as the DNA-directed RNA polymerase. The abundance of hydrogenase genes increased with depth from 0 to 129 m. Hydrogenases were present in 481 out of 1245 metagenome-assembled genomes. The relative abundance of microbes with hydrogenases accounted for ~50% of the entire community. Hydrogenases were actively expressed, making up as much as 5.9% of methanogen proteomes. Most of the newly discovered diversity of hydrogenases was in “Group 3b”, which has been associated with sulfur metabolism. “Group 3d”, facilitating the interconversion of electrons between hydrogen and NAD, was the most abundant and mainly observed in methanotrophs and chemoautotrophs. “Group 3a”, associated with methanogenesis, was the most abundant in proteomes. Two newly discovered groups of [NiFe]-hydrogenases, observed in *Methanobacteriaceae* and *Anaerolineaceae*, further expanded diversity. Our results highlight the vast diversity, abundance and expression of hydrogenases in groundwaters, suggesting a high potential for hydrogen oxidation in subsurface habitats.

Generating hydrogen (H_2_) from solar and wind energy, and subsequently storing it on a terawatt scale in the subsurface is currently considered a key aspect of the energy transition [1-3]. One of the potential challenges of this approach is the microbial oxidation of hydrogen, which could induce hydrogen loss [4-6]. Our recent work suggested a high potential for microbial hydrogen turnover in groundwaters, based on dissolved hydrogen concentrations, as well as detection and activity of hydrogenotrophic methanogens [7]. Here we explored the diversity and potential functions of hydrogenases with an expanded sample set encompassing 265 groundwater samples (geochemically characterised and amplicon sequenced, with additional 25 metagenomes and 5 proteomes) from 138 wells in Alberta (Canada), with sampling depths between 0 and 157 m (Fig. S1, Tables S1, S2). The groundwaters displayed a range of oxidation states from oxic to completely reduced, accompanied with a wide range of sulfate (>10 g/L to below detection) and methane concentrations (74 mg/L to below detection).

The abundance and expression of different types of hydrogenases were estimated based on unassembled reads, assembled contigs, metagenome-assembled genomes (MAGs), and proteins in 25 groundwaters. Few [Fe]- and [FeFe]-hydrogenases were present in our data. Notably, the catalytic subunit of [NiFe]-hydrogenase was as abundant as the DNA-directed RNA polymerase (*rpoB*) (Fig. 1A, Table S3). In 12 out of 25 metagenomes, hydrogenase genes were more abundant than *rpoB* genes, indicating multiple copies of same subtypes or various subtypes of hydrogenases in a single genome. The ratio of hydrogenase over *rpoB* correlated positively with depth (*P* = 0.009, Fig. 1B). From metagenomes, 616 high-quality and 629 medium-quality MAGs were obtained. Hydrogenases were present in 481 out of 1245 MAGs, which together accounted on average for 50% of the relative abundance of all MAGs (Fig. 1C, Table S4). In eight samples, MAGs with hydrogenases accounted for >70% of all MAGs. Although conducting proteomics with groundwater samples is challenging due to low cell counts, we obtained proteomes of five groundwaters, showing hydrogenases accounted for 0.016–1.0% of all proteins (Tables S5–S9). In proteomes of individual species associated with our MAGs, the relative abundance of hydrogenases ranged from 0.0026% (*Methylomonadaceae*) to 5.9% (*Methanobacteriaceae*) (Fig. 1D). Interestingly, hydrogenase was more abundant in the three methanogen proteomes (>1.3%) than the 21 bacterial proteomes (<0.19%) (Tables S5–S9). Thus, hydrogenase genes might be one of the most prevalent genes in the subsurface and active expression indicated these genes were functional.

**Figure 1:**
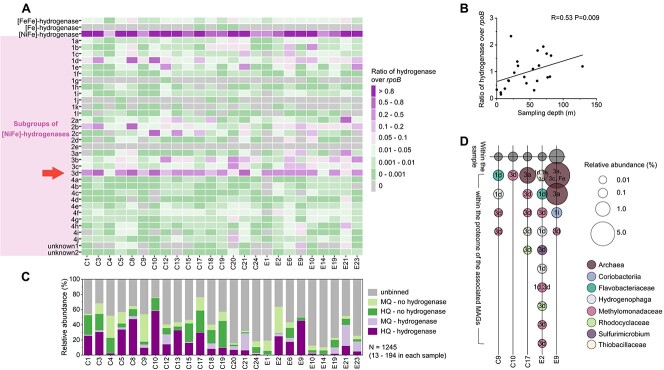
Abundance and expression of hydrogenases. (A) Ratio of reads mapped to hydrogenase genes over reads mapped to *rpoB* genes in metagenome of 25 groundwater samples. The arrow indicates the subgroup of hydrogenases with the highest abundance in sampled groundwaters. (B) Relationship between depth and total abundance of hydrogenases. Spearman’s rank correlation coefficient and the *P*-value are shown. The line shows the linear regression. (C) Relative abundance of 1245 MAGs (13–194 per sample) with and without hydrogenases. MQ: medium-quality. HQ: high- quality. Relative abundances were based on reads mapped to an MAG divided by total reads mapped. (D) Abundance of hydrogenases in proteomes. Relative abundance within a sample was calculated as % of all peptide spectral matches of the sample. Relative abundance for individual MAGs was calculated as % of all peptide spectral matches associated with the MAG.

From the assembled contigs, 1545 [NiFe]-hydrogenase gene sequences were recovered (Supplementary Result 1), which displayed vast diversity (Table S10). These groundwater hydrogenase sequences considerably increased the number (1999) of [NiFe]-hydrogenase sequences present in a widely used database (Fig. 2) [8]. The newly discovered diversity, abundance, and expression were concentrated among a few specific subtypes of [NiFe]-hydrogenases, groups 1e, 3a, 3b, and 3d. Most of the new diversity was observed in group 3b, while group 3d was the most abundant in metagenomes and group 3a was the most abundant in proteomes.

**Figure 2:**
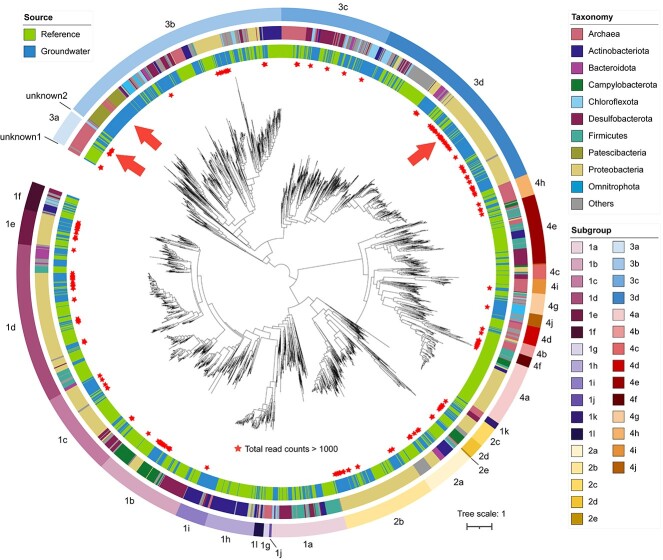
Phylogenetic tree of the catalytic subunit of [NiFe]-hydrogenases. The tree is midpoint-rooted. An arrow inside indicates the place of [NiFe]-hydrogenases with high diversity, abundance, or expression discovered in sampled groundwaters. Any sequences with total read counts over 1000 in the 25 samples are marked with a star. From inside to outside, the three rings around the tree indicate (1) source, (2) phylum-level taxonomy, and (3) subgroups based on HydDB [5].

Hydrogenases of groups 1e and 3b are associated with sulfur reduction [8, 9]. In our data, the abundance of groups 1e and 3b both positively correlated with sulfate concentration (*P* = 0.025 and 0.007, respectively, Fig. S2). Group 3b hydrogenases were occasionally observed in close proximity to sulfhydrogenase subunit delta and sulfite reductase subunit A (Tables S12, S13). Among high-quality MAGs, group 1e was exclusively present in members of Burkholderiales, and sometimes co-existed with group 3b. Group 3b was commonly detected in MAGs of sulfate-reducing microorganisms, particularly thirteen Desulfobacterota and three Thermodesulfovibrionia. These MAGs also encoded sulfate adenylyltransferase, adenylylsulfate reductase, and dissimilatory sulfite reductase (Table S11). Some group 3b hydrogenases were detected in MAGs of sulfur-oxidising microorganisms such as three *Gallionellaceae* that encoded sulfide:quinone oxidoreductase and sulfite dehydrogenase, and four *Thiobacillaceae* that contained the thiosulfate oxidation sox complex. These hydrogenases might also function alongside sulfur oxidation, coupled to oxygen or nitrate reduction. Many other group 3b were detected in genomes of microbial “dark matter” clades, such as Patescibacteria (14) and Omnitrophota (5), consistent with previous findings [10, 11].

Group 3a hydrogenases are associated with methanogenesis [8, 9] and were most abundant in our proteomes (Fig. 1D, Tables S5–S9). They were exclusively observed in MAGs of hydrogenotrophic methanogens, six Methanomicrobiales and four Methanobacteriales [12]. All Methanobacteriales also encoded tetrahydromethanopterin-reducing [Fe]-hydrogenases. Nine of them possessed tetrahydromethanopterin S-methyltransferase (Mtr), the key enzyme for hydrogenotrophic methanogenesis [13]. Two of them contained Methylamine:Coenzyme M Methyltransferase (mtbA), suggesting that they might produce methane from methylamine [14]. These results were consistent with previous research showing active conversion of CO_2_ into methane in hydrocarbon reservoirs [15].

Group 3d is associated with fermentative metabolism and chemoautotrophy, interconverting electrons between hydrogen and NADH depending on cellular redox state [8, 9]. Group 3d was the most abundant subgroup in 15 out of 25 groundwater samples (Fig. 1A). Most group 3d hydrogenase genes were close to an NADP oxidoreductase gene (Tables S13, S14). 3d hydrogenase genes were present in 89 high-quality MAGs, with 12 of them encoding formate C-acetyltransferases or lactate dehydrogenases, both signature genes of fermentative metabolism. Of these MAGs, 21 were associated with methanotrophic Methylomonadaceae. For the other 68 MAGs, 40 of them contained both RuBisCO and phosphoribulokinase, indicating a functional Calvin cycle. For instance, MAGs associated with *Rhodocyclaceae* (12), *Hydrogenophaga* (7), *Nitrosomonas* (7), and *Rhodoferax* (5) fall into this category. Thus, it is likely that these chemolithoautotrophs can use hydrogen as an additional energy source, with the hydrogenase transferring electrons from H_2_ to NAD^+^ to drive their Calvin cycles.

Two newly discovered groups of [NiFe]-hydrogenases further expanded diversity. The first was positioned near the root of the tree (Fig. 2). This group consisted of three sequences, exclusively found in *Methanobacteriaceae*. The other newly discovered group was near the root of group 3b, composed of six sequences, including five sequences affiliated with *Anaerolineaceae* and one affiliated with Bathyarchaeia.

Consistency in the types/subgroups of hydrogenases and metabolisms among MAGs with the same taxonomic identity was observed for common groundwater residents, which helped to extrapolate metagenomic findings to 265 amplicon-sequenced groundwater samples. For example, the total relative abundance of *Methylomonadaceae* bacteria (all 21 MAGs had hydrogenases) could reach 88.6% (Table S15). Members of *Hydrogenophaga* (8 out of 14 MAGs had hydrogenases) could be as abundant as 71.2%. These findings suggest a high potential for hydrogen consumption in sampled subsurface habitats.

While the subsurface ecosystems analysed here would not be suitable for hydrogen storage, our study adds to growing evidence that hydrogenases are diverse, functional and ubiquitous in subsurface environments [16-19]. However, as hydrogenases were most abundant in methanogen proteomes, this need not always be a barrier to hydrogen storage, since recovery of methane could still be a desirable outcome. Likely, any subsurface environment at a temperature conducive to life would harbor microorganisms that thrive on hydrogen.

## Supplementary Material

SupplementaryInformation_ycae023

SupplementaryTables_ycae023

SupplementaryResult1_NiFe-hydrogenases_groundwater_ycae023

SupplementaryResult2_NiFe-hydrogenases_ref_gw_alignments_ycae023

## Data Availability

Amplicons in this study are under the Bioproject PRJNA861683 and PRJNA700657. Metagenomes and metagenome-assembled genomes are under the Bioproject PRJNA700657 (NCBI). The mass spectrometry proteomics data have been deposited to the ProteomeXchange Consortium via the PRIDE partner repository [20] with the dataset identifier PXD044305.

## References

[1] Krevor S , de Coninck H, Gasda SE et al. Subsurface carbon dioxide and hydrogen storage for a sustainable energy future. Nat Rev Earth Environ 2023;4:102–18. 10.1038/s43017-022-00376-8.

[2] Tarkowski R . Underground hydrogen storage: characteristics and prospects. Renew Sust Energ Rev 2019;105:86–94. 10.1016/j.rser.2019.01.051.

[3] Zivar D , Kumar S, Foroozesh J. Underground hydrogen storage: a comprehensive review. Int J Hydrogen Energ 2021;46:23436–62. 10.1016/j.ijhydene.2020.08.138.

[4] Momper L , Jungbluth SP, Lee MD et al. Energy and carbon metabolisms in a deep terrestrial subsurface fluid microbial community. ISME J 2017;11:2319–33. 10.1038/ismej.2017.94. 28644444 PMC5607374

[5] Stevens TO , McKinley JP. Lithoautotrophic microbial ecosystems in deep basalt aquifers. Science 1995;270:450–5. 10.1126/science.270.5235.450.

[6] Lappan R , Shelley G, Islam ZF et al. Molecular hydrogen in seawater supports growth of diverse marine bacteria. Nat Microbiol 2023;8:581–95. 10.1038/s41564-023-01322-0. 36747116 PMC10305171

[7] Ruff SE , Humez P, de Angelis IH et al. Hydrogen and dark oxygen drive microbial productivity in diverse groundwater ecosystems. Nat Commun 2023;14:3194. 10.1038/s41467-023-38523-4. 37311764 PMC10264387

[8] Sondergaard D , Pedersen CN, Greening C. HydDB: a web tool for hydrogenase classification and analysis. Sci Rep 2016;6:34212. 10.1038/srep34212. 27670643 PMC5037454

[9] Greening C , Biswas A, Carere CR et al. Genomic and metagenomic surveys of hydrogenase distribution indicate H_2_ is a widely utilised energy source for microbial growth and survival. ISME J 2016;10:761–77. 10.1038/ismej.2015.153. 26405831 PMC4817680

[10] Alvarado LEV , Fakra SC, Probst AJ et al. Autotrophic biofilms sustained by deeply-sourced groundwater host diverse CPR bacteria implicated in sulfur and hydrogen metabolism. Microbiome 2024;12:15. 10.1186/s40168-023-01704-w. 38273328 PMC10811913

[11] Williams TJ , Allen MA, Berengut JF et al. Shedding light on microbial "dark matter": insights into novel Cloacimonadota and Omnitrophota from an Antarctic lake. Front Microbiol 2021;12:741077. 10.3389/fmicb.2021.741077. 34707591 PMC8542988

[12] Thauer RK , Kaster AK, Goenrich M et al. Hydrogenases from methanogenic archaea, nickel, a novel cofactor, and H_2_ storage. Annu Rev Biochem 2010;79:507–36. 10.1146/annurev.biochem.030508.152103. 20235826

[13] Berghuis BA , Yu FB, Schulz F et al. Hydrogenotrophic methanogenesis in archaeal phylum Verstraetearchaeota reveals the shared ancestry of all methanogens. Proc Natl Acad Sci U S A 2019;116:5037–44. 10.1073/pnas.1815631116. 30814220 PMC6421429

[14] Vanwonterghem I , Evans PN, Parks DH et al. Methylotrophic methanogenesis discovered in the archaeal phylum Verstraetearchaeota. Nat Microbiol 2016;1:16170. 10.1038/nmicrobiol.2016.170. 27694807

[15] Tyne RL , Barry PH, Lawson M et al. Rapid microbial methanogenesis during CO_2_ storage in hydrocarbon reservoirs. Nature 2021;600:670–4. 10.1038/s41586-021-04153-3. 34937895 PMC8695373

[16] Hernsdorf AW , Amano Y, Miyakawa K et al. Potential for microbial H2 and metal transformations associated with novel bacteria and archaea in deep terrestrial subsurface sediments. ISME J 2017;11:1915–29. 10.1038/ismej.2017.39. 28350393 PMC5520028

[17] Adhikari RR , Glombitza C, Nickel JC et al. Hydrogen utilization potential in subsurface sediments. Front Microbiol 2016;7:8. 10.3389/fmicb.2016.00008. 26858697 PMC4726784

[18] Hidalgo KJ , Sierra-Garcia IN, Zafra G et al. Genome-resolved meta-analysis of the microbiome in oil reservoirs worldwide. Microorganisms 2021;9:1812. 10.3390/microorganisms9091812. 34576708 PMC8465018

[19] Vigneron A , Alsop EB, Lomans BP et al. Succession in the petroleum reservoir microbiome through an oil field production lifecycle. ISME J 2017;11:2141–54. 10.1038/ismej.2017.78. 28524866 PMC5563965

[20] Perez-Riverol Y , Csordas A, Bai J et al. The PRIDE database and related tools and resources in 2019: improving support for quantification data. Nucleic Acids Res 2019;47:D442–50. 10.1093/nar/gky1106. 30395289 PMC6323896

